# Understanding the common mechanisms of heart and skeletal muscle wasting in cancer cachexia

**DOI:** 10.1038/s41389-020-00288-6

**Published:** 2021-01-08

**Authors:** Valentina Rausch, Valentina Sala, Fabio Penna, Paolo Ettore Porporato, Alessandra Ghigo

**Affiliations:** 1 Department of Molecular Biotechnology and Health Sciences, Molecular Biotechnology Center, University of Torino, Torino, Italy; 2 Department of Clinical and Biological Sciences, University of Torino, Torino, Italy

**Keywords:** Metabolism, Autophagy, Extracellular signalling molecules, Proteolysis

## Abstract

Cachexia is a severe complication of cancer that adversely affects the course of the disease, with currently no effective treatments. It is characterized by a progressive atrophy of skeletal muscle and adipose tissue, resulting in weight loss, a reduced quality of life, and a shortened life expectancy. Although the cachectic condition primarily affects the skeletal muscle, a tissue that accounts for ~40% of total body weight, cachexia is considered a multi-organ disease that involves different tissues and organs, among which the cardiac muscle stands out for its relevance. Patients with cancer often experience severe cardiac abnormalities and manifest symptoms that are indicative of chronic heart failure, including fatigue, shortness of breath, and impaired exercise tolerance. Furthermore, cardiovascular complications are among the major causes of death in cancer patients who experienced cachexia. The lack of effective treatments for cancer cachexia underscores the need to improve our understanding of the underlying mechanisms. Increasing evidence links the wasting of the cardiac and skeletal muscles to metabolic alterations, primarily increased energy expenditure, and to increased proteolysis, ensuing from activation of the major proteolytic machineries of the cell, including ubiquitin-dependent proteolysis and autophagy. This review aims at providing an overview of the key mechanisms of cancer cachexia, with a major focus on those that are shared by the skeletal and cardiac muscles.

## Introduction

Cachexia is a devastating syndrome, often announcing the onset of the terminal phase of several diseases, including respiratory and cardiac failure, AIDS, sepsis as well as cancer. It is defined as an unstoppable weight loss of at least 5% of body mass in 6 months, mostly affecting lean mass, while fat tissue wasting occurs at a variable penetrance. In some cases, cachexia might be masked by concomitant obesity, in which loss of lean mass is counteracted by fat deposition^1^. Cachexia occurs in at least 80% of metastatic cancer patients, thus representing a highly penetrant complication and the primary cause of death in at least one-third of cancer patients. To date, cachexia still represents an unmet medical need, because a substantial portion of patients suffering from chronic diseases succumb to this complication, due to the lack of therapeutic options.

**Fig. 1:**
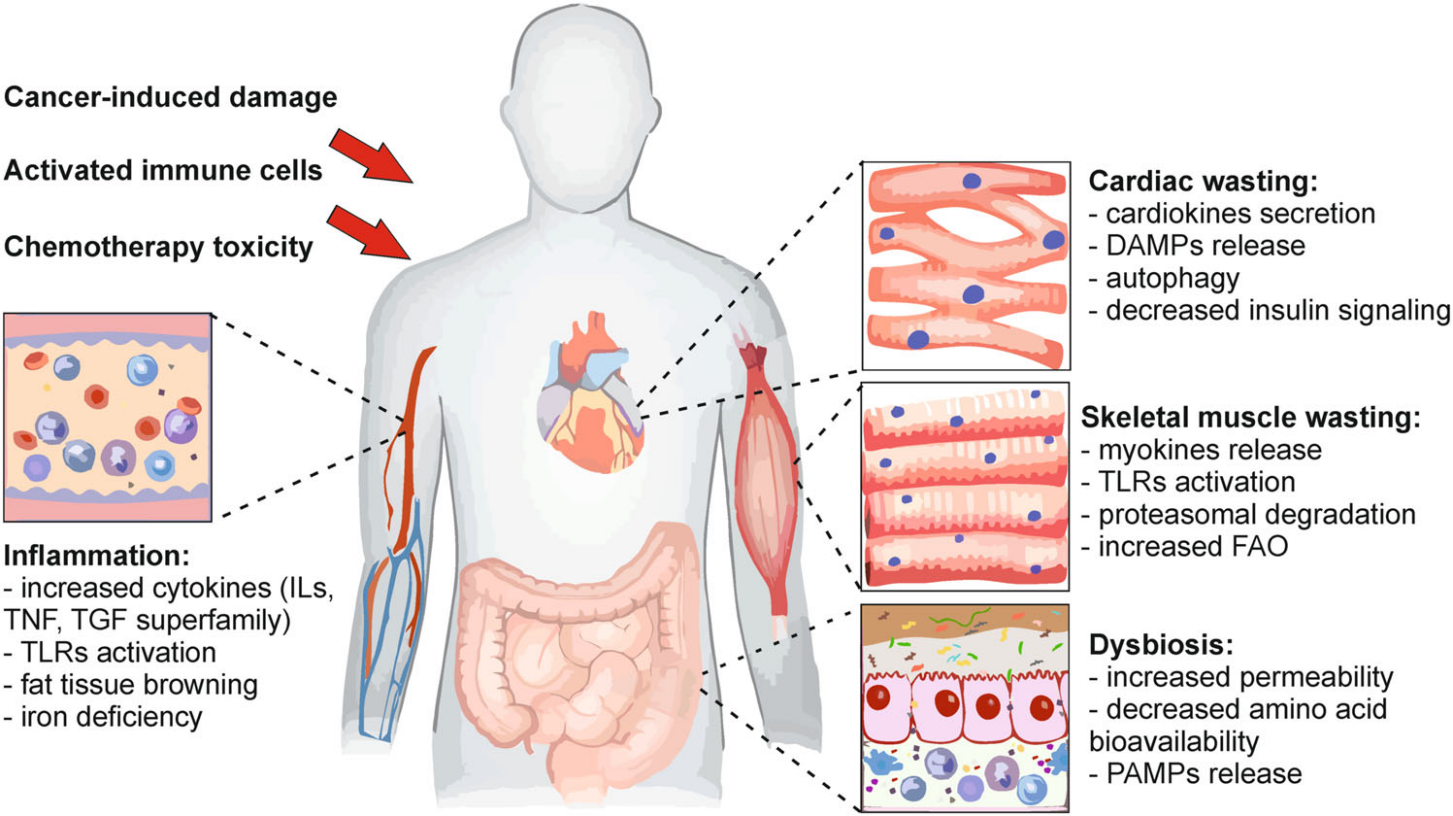
Multi-organ alterations in cancer-induced cachexia. Cancer cells, together with the activation of the inflammatory response and the toxic effects of chemotherapy, contribute to concomitant and interconnected alterations in multiple distant organs, including the cardiac and skeletal muscles and the gut, in the course of cancer-induced cachexia. ILs interleukins, TNF tumor necrosis factor, TGF transforming growth factor, TLRs toll-like receptors, DAMPs damage-associated molecular patterns, FAO fatty acid oxidation, PAMPs pathogen-associated molecular patterns.

Despite its major burden on life quality and healthcare systems, our knowledge of the disease is still limited. Cachexia is indeed a complex syndrome affecting several organs, promoting systemic metabolic rewiring, and a diffuse inflammatory condition. Cachectic patients present increased resting energy expenditure, mostly due to systemic lipolysis and mitochondrial dysfunction, while systemic inflammation contributes to local tissue dysfunctions such as anorexia and fat tissue browning. Furthermore, cachexia is often associated with gut dysbiosis and intestinal membrane permeabilization, resulting in elevated levels of circulating proinflammatory molecules that further worsen systemic inflammation^2^ (Fig. 1).

Nevertheless, the major manifestation of cachexia is skeletal muscle wasting, which results in lean mass loss and frailty. Importantly, muscle wasting results in a severe drop in quality of life, causing respiratory distress and fatigue^3^. Moreover, muscle mass loss is an important sign of suffering, hence many pharmacological regimens are normally interrupted once systemic weight loss occurs.

During cachexia, cardiac wasting can also occur, primarily as a consequence of cardiac proteins loss^2^. This is for example the case of cancer patients where cardiac wasting is often secondary to therapy with cardiotoxic anti-cancer drugs or the presence of the tumor that, by producing circulating mediators, promotes atrophy of cardiomyocytes and negatively impacts on cardiac contractility^4^. Vice versa, cardiac dysfunction itself promotes skeletal muscle wasting, a complication known as cardiac cachexia. Furthermore, cardiac cachexia is an independent predictor of survival in chronic heart failure patients^5^ in experimental models^6–9^ and in up to 19.5% of heart failure patients, particularly those with reduced ejection fraction^10^.

Understanding the complex crosstalk between the heart, the skeletal muscle, and the host in chronic disease conditions, particularly in cancer, is of utmost importance for the identification of novel potential targets for therapeutic approaches. Here, we will discuss the current knowledge of the common biological basis of muscle and heart wasting, with particular reference to cancer-induced cachexia.

## Inter- and intracellular mediators of skeletal and heart muscle cachexia

Skeletal muscle and cardiac wasting has been demonstrated in some cancer types, including lung, pancreatic, and gastrointestinal tumors^11^. Although cancer cells rarely metastasize to skeletal and cardiac muscle, factors secreted by either the primary tumor, metastases, or activated immune cells can induce extensive muscle wasting. Different from other types of muscle atrophy, like those induced by fasting, denervation, or disuse, cancer cachexia is characterized by massive systemic inflammation^12,13^. Cytokines and other pro-cachectic mediators can be directly released by some types of cancer cells into the bloodstream, however the majority of catabolic cytokines is generated by immune cells in response to cancer^14^. Furthermore, organ damage, induced by metastatic erosion or chemo- and radiotherapy, may lead to the secretion of danger-associated molecular patterns (DAMPs), endogenous signals of cell damage^15^ that contribute to inflammation and the development of the cachectic syndrome. Finally, both cardiac and skeletal muscle can act as endocrine organs, by releasing signaling molecules called myokines and cardiokines, respectively, which include members of the transforming growth factor (TGF) superfamily (like Myostatin and Activin A)^16^. All these circulating mediators are involved in the promotion of skeletal and/or cardiac muscle catabolism and convey the pro-atrophic signals that trigger cancer cachexia.

**Fig. 2:**
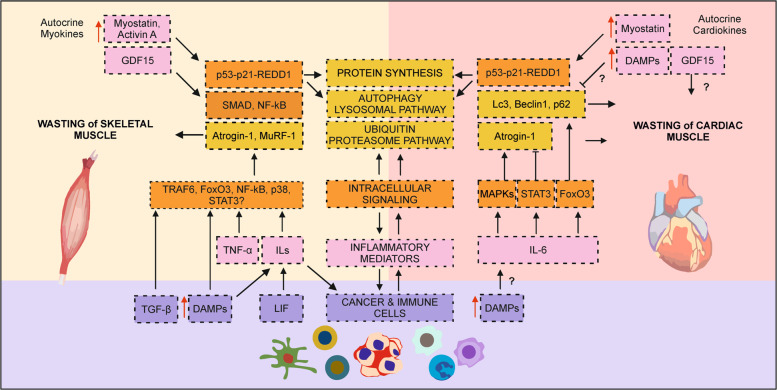
Pathological alterations underlying muscle wasting in cancer cachexia. The mechanisms underlying cancer cachexia are multiple and intertwined. Either factors released by skeletal and cardiac muscles (myokines and cardiokines respectively; pink boxes) or factors secreted by cancer and cancer-associated immune cells (violet boxes) trigger a cascade of processes which ultimately result in cachexia. The circulating factors (pink), intracellular signaling pathways (orange), and final effectors of wasting (yellow) that lead to skeletal (left side of the figure) and cardiac (right side of the figure) muscle wasting are reported. Albeit the biological processes underlying skeletal and cardiac muscle wasting are similar, their relative contribution and the specific molecular players involved differ slightly. Red arrows indicate those factors that are increased as a consequence of chemotherapy. DAMPs damage-associated molecular patterns, GDF15 growth differentiation factor 15, ILs interleukins, LIF leukemia inhibitory Factor, TNF-α tumor necrosis factor alpha, TGF-β transforming growth factor beta.

At the intracellular level, factors released by the tumor, its environment, or activated immune cells mediate the activity of a large variety of signaling molecules, like NF-κB, p38 MAPK, or STAT3, orchestrating inter- and intracellular signaling that ultimately promote cancer cachexia^17–22^ (Fig. 2). Among those are pathways controlling protein degradation, including the autophagy-lysosomal pathway (ALP) and the ubiquitin-proteasome pathway (UPP)^13^, whose alteration is a major hallmark of cancer cachexia. Indeed, in cancer cachexia, ALP and UPP are hyperactive, leading to muscle atrophy^13,23,24^.

### Autophagy-lysosomal pathway and ubiquitin-proteasome pathway

The negative protein balance observed in the wasting syndrome is linked to protein hypercatabolism. Both ALP and UPP efficiently degrade proteins that have been ubiquitylated by E1, E2, and E3 enzymes^25^. In ALP, uniquely ubiquitylated proteins are engulfed by autophagosomes that subsequently fuse to lysosomes to form autolysosomes, where proteins are enzymatically degraded^25,26^. On the contrary, differently ubiquitylated proteins are recognized by the UPP and degraded by the proteasome^27^. Increasing evidence indicates that the contribution of ALP and UPP to muscle wasting is context-dependent, varying between pathologies^13^. For example, the muscle-specific E3 ubiquitin ligases Atrogin-1 and MuRF-1 are the main drivers of skeletal but not cardiac muscle wasting, in which autophagy has instead a major role^28^. This might be explained by the fact that the heart has a higher metabolic rate and protein turnover than the skeletal muscle. Thus, induction of ALP, in the presence of a high basal activity of UPP, might be sufficient to mediate protein degradation^28^. On the other hand, both UPP and ALP contribute to skeletal muscle wasting. Accordingly, direct comparison between skeletal and cardiac muscle in a cachectic rat model showed the upregulation of autophagy markers, such as LC3 and p62, in both tissues. In contrast, TRAF6, an inducer of atrophy, and Beclin1, an autophagic marker, were specifically upregulated in the gastrocnemius and the heart, respectively^29–31^. In line with this observation, cachectic mice exhibited atrophic hearts, with enhanced expression of Beclin1 and LC3 but no significant induction of proteins involved in ubiquitination or apoptosis^28,32^. Intriguingly, forkhead box transcription factors 3 (FoxO3), which is an established inducer of proteasomal-mediated atrophy in skeletal muscle^1^, has been reported to induce atrophy via ALP in the heart^33^. On the other hand, cardiac atrophy, in majorly cancer-independent disease models, is accompanied by increased levels of Atrogin-1 and therefore an active UPP, which results from the induction of MAPK pathways^34–36^. One study on tumor-bearing mice reports the increase of atrogenes expression in the heart^37^ and indicates the role of UPP in cancer cachexia which remains to be further defined.

Consequently, the induction of the different molecular pathways that cause atrophy in skeletal and the cardiac muscles^32^ may depend on the different composition of humoral factors that are released by the tumor or cancer-affected tissues.

### The inflammatory milieu

Cancer cachexia is accompanied by an increased release of inflammatory molecules, which are mainly produced by immune cells in response to cancer^38^. Among these are interleukins, tumor necrosis factor, and members of the transforming growth factor family.

#### Interleukins

Levels of IL-6 cytokine family members are increased during cancer-related cachexia^39^, as a consequence of the release of leukemia inhibitory factor (LIF) from tumor cells^40^. IL-6, in turn, induces intracellular STAT3 (detailed in Box 1), p38, and FoxO signaling in skeletal muscles^21,39^ through Glycoprotein 130^39^. These are common intracellular signaling pathways elicited by inflammatory cytokines like IL-1^41^ and IL-8^42^. Interestingly, the loss of cardiac mass in tumor-bearing mice does not rely on circulating levels of LIF^40^.

BOX 1 STAT3. Signal transducer and activator of transcription-3 (STAT3) transduces signals from receptors and intracellular kinases in order to regulate gene transcription^194^. Among others, STAT3 is activated by the IL-6/GP-130/JAK pathway, with IL-6 being a main promoter of cachexia (see review^21,195^ for details). Induction of STAT3 in myofibers, in turn, leads to the expression of pro-atrophic genes^196,197^. However, the investigation of this pathway in clinical samples is challenging and requires further research^195^. Therefore, despite strong experimental indications, the role of IL-6-mediated induction of STAT3 activity remains to be proven in cancer cachexia patients^195^. Unlike in skeletal muscles, in the heart STAT3 preserves cardiac function and size^195^. Accordingly, STAT3 deficiency was associated with declined cardiac contractility, microtubule instability, and disruption of UPP in cardiomyocytes^195,198–200^. In the light of its different implications in skeletal and heart muscle^195^, further studies are required to determine the muscle-specific role of STAT3 in cancer cachexia.

**Tumor necrosis factor**

The first inflammatory cytokine to be linked to cancer cachexia was the tumor necrosis factor (TNF), also known as cachectin, due to its elevation in the blood of cachectic cancer patients and its capacity to induce muscle wasting in animal models^43^. TNF-α activates NF-κB (detailed in Box 2), which leads to muscle wasting and reduced muscle regeneration^44,45^.

Recently, the cachectic capacity of TNF-α was linked to the upregulation of the zinc importer ZRT- and IRT-like protein 14 (ZIP14) in the wasting muscles of mice and patients with metastatic cancer^46^. The increase in ZIP14 is responsible for zinc accumulation in cachectic muscles, blocks muscle cell differentiation, and causes myosin heavy chain loss, overall contributing to muscle atrophy and weakness^46^. ZIP14 upregulation and altered zinc homeostasis are major underlying features of cachexia related to pancreatic cancer^47^. Of note, both skeletal and cardiac muscle catabolism occur in pancreatic cancer mouse models^48^ and patients^11^. In the heart, ZIP14 is expressed at relatively high levels^49^, and is increased after doxorubicin treatment^50^, resulting in increased intracellular zinc levels and induction of sarcoplasmic reticulum stress^51^.

BOX 2 NF-kB. NF-κB and TNF-α interact in a positive feedback loop^201,202^, while TNF-α activation induces MAPKs, like p38, which further induce atrophic genes such as Atrogin-1^20^. NF-κB has been widely studied in the context of skeletal muscle atrophy, yet little is known about the function of NF-κB in heart muscle wasting. However, activation of NF-κB leads to cardiomyocyte atrophy in Duchenne muscular dystrophy, indicating that it potentially plays a role also in heart muscle wasting^203^.

**The transforming growth factor superfamily**

Among the stimuli leading to ZIP14 upregulation is also TGF-β^47^, one of the members of the TGF superfamily^52^. Many tumors show increased expression of TGF superfamily members, which can be further enhanced by chemotherapy^52^. For example, TGF-β, which is implicated in the metabolic changes associated with cancer cachexia^51^, is released from the bone as a result of metastasis-induced bone destruction^53^.

Two other members of the TGF superfamily, Myostatin and Activin A, negatively regulate muscle mass by binding to the Activin II B Receptor (ACVR2B). Myostatin, also known as Growth Differentiation Factor (GDF) 8, impairs satellite cell activation, myoblast proliferation and differentiation^54,55^ as well as it promotes muscle loss^56^. Consistently, muscle Myostatin levels are increased in experimental cancer-induced cachexia^57^. Interestingly, Myostatin is also a cardiokine that is expressed and secreted by the myocardium during end‐stage heart failure^58^. In accord with its catabolic effects, Myostatin released from the failing myocardium is responsible for the induction of skeletal muscle atrophy in experimental models^59^.

Similarly, p38-mediated activation of ACVR2B by Activin A induces catabolic effects in the muscle^19^ (detailed in Box 3). Of note, circulating Activin A levels are an independent predictor of survival in cancer patients^60^. Consistently, blockage of ACVR2B abolishes the activation of UPP and the induction of atrophy-specific ubiquitin ligases in muscles, stimulates muscle stem cell growth, and reverses prior loss of skeletal muscle and cancer-induced cardiac atrophy^61^, even in the presence of anti-cancer therapies^62^.

Intriguingly, doxorubicin itself increases the expression of Myostatin in skeletal muscle^63^. Accordingly, doxorubicin‐induced cachexia is mediated by the activation of a common p53-p21-REDD1 pathway in both skeletal and cardiac muscles and can be prevented by ACVR2B ligand blocking. Notably, treatment with soluble ACVR2B‐Fc decoy receptor has a minor impact on the heart compared to skeletal muscles^64^, suggesting that ACVR2B blockage is an appealing strategy for reducing cancer-induced wasting of skeletal and, to a lesser extent, cardiac muscle. Consequently, several strategies targeting the ACVR2 pathway are under evaluation in clinical trials to treat pathological muscle loss and weakness^65–68^.

Furthermore, GDF11, ligand of ACVR2B and highly homologous to Myostatin, is involved in the promotion of striated muscle catabolism^69^, since supraphysiological levels of GDF11 induce cardiac and skeletal muscle dysfunction and wasting^70–72^. Moreover, GDF11 increases plasma levels of Activin A and GDF15, another distant member of the TGF-β superfamily^73^, which further contribute to anorexia-cachexia syndrome.

GDF15, also known as MIC-1, has been implicated in cancer cachexia^74^, heart failure-induced cachexia^9^, and systemic energy metabolism^75^. GDF15 is both a myokine^75,76^ and a cardiokine^9,77^. It is produced by muscle cells and secreted into the bloodstream, acting on distant target organs through binding to the GDNF-family receptor α-like (GFRAL) receptor^78–80^. Circulating GDF15 levels correlate with weight loss^81^ and poor survival^82^ in cancer patients and are increased early after tumor injection in models of cancer cachexia, in which GDF15 is implicated in MuRF-1 activation and atrophy^83^ as well as in inducing anorexia and emesis, further worsening the negative energy balance^84,85^.

BOX 3 P38. The p38 MAP kinase regulates transcription, chromatin remodeling, cytoskeletal dynamics, and protein degradation^204^. p38 is also required for muscle differentiation as it regulates MyoD activity and Myogenin expression^204–207^. In tumor-bearing mice, the inhibition of p38 activity facilitates protein ubiquitination through upregulation of Atrogin-1 and, possibly, MuRF-1 expression^206^. Furthermore, p38 mediates the activation of the receptor ACVR2B after Activin A binding. This interaction results in the upregulation of ubiquitin ligases Atrogin-1 and UBR2 (E3αII) and of the autophagosome marker LC3II^19^. Although the role of p38 in skeletal muscle is well-described, little is known about its involvement in the cardiac muscle during cancer cachexia. However, p38 inhibition leads to the induction of growth factor expression in the adult myocardium^208^. Furthermore, an increased activity of p38 has been observed in both animal models and patients with heart failure, indicating a yet to be fully discovered role of p38 in the malfunctioning heart^209^.

### Danger-associated molecular patterns and toll-like receptors

DAMPs are part of a plethora of molecules^3^, including free and histone-associated nuclear DNA^86^, mitochondrial DNA (mtDNA)^87^, and heat shock proteins^88^, which can be released by cancer, immune as well as cardiac cells upon injury. DAMPs are the endogenous agonists of Toll-Like Receptors (TLRs), an evolutionarily ancient family of pattern recognition receptors^89^. In immune cells, plasma membrane TLRs stimulate the synthesis of proteins that belong to the inflammasome complex, by inducing the translocation of NF-κB into the nucleus, ultimately modulating innate immunity^90^. In muscle cells, NF-κB activation is sufficient to induce mass loss through the upregulation of E3 ubiquitin ligase MuRF-1, e.g. upon LPS-induced pulmonary inflammation^91^ or LLC-derived tumor in mice^92^.

In cancer cachexia, the activation of TLRs by DAMPs, released in the bloodstream, stimulates muscle proteolysis both directly, by acting on muscle cells, and indirectly, by activating TLR4 in immune cells to increase systemic inflammation^88^. Indeed, TLR4 is the isoform which is mainly linked to muscle wasting in cancer, being required for LLC-cancer-related muscle wasting^93,94^. Accordingly, the TLR4 expression level in skeletal muscles of cancer patients significantly correlates with low skeletal muscle index and weight loss^95^. Interestingly, the role of TLRs in cancer-induced muscle catabolism is relatively isoform- and disease-specific. For instance, muscle-specific activation of TLR7 by tumor-secreted microvesicles promotes skeletal muscle cell death^96,97^, while local activation of TLR7 in the tumor stroma triggers CD8^+^ T-cells, resulting in tumor shrinkage and, consequently, in reduced cachexia and improved survival^98^.

Although, to date a clear link between cancer cachexia and TLRs is missing, it is plausible that the same pathway may be detrimental for the heart. For instance, activation of specific TLR isoforms expressed by cardiac cells has been linked to pro-inflammatory effects, with TLR2, TLR4, and TLR5 being responsible for NF-κB-dependent induction of the inflammasome^99,100^. The inflammasome complex, in turn, may initiate the activation of pro-inflammatory cascades, leading to pyroptotic cell death^101^, as in the case of acute myocardial infarction^102^.

### Metabolic changes underlying skeletal and heart muscle wasting induced by cancer

From the biochemical standpoint, cachexia is considered a metabolic disease linked to the negative energy balance between calorie intake and dissipation, which eventually promotes systemic wasting and body weight loss. Such an imbalance is, on the one side, due to the loss of appetite and reduced nutrient absorption, and, on the other side, a result of the upregulation of energy-consuming processes and metabolic dysfunction, which collectively increase the energetic needs of the body driving calorie wasting.

Insufficient calorie intake is mostly driven by anorexia, a persistent and unphysiological loss of appetite. All chronically ill patients develop various degrees of anorexia, due to depression and neuroinflammation. The so-called sick state, driven by systemic inflammation, has also been proposed as a conserved evolutionary mechanism to limit nutrient availability during infections, in order to restrain nutrient availability to pathogens. Consistently, cachexia is often referred to as CAC (cachexia and anorexia) syndrome.

Recently, it has been demonstrated that cachectic patients experience impaired intestinal function and absorption, which is at least in part caused by the alteration of the gut microbiome^103,104^.

Collectively, reduced calorie intake and nutrient uptake drive a systemic energetic failure. Nevertheless, restoring proper nutrient supply is not sufficient to recover body mass homeostasis, but only delays wasting progression^1^. Such evidence highlights that other mechanisms, like increased calorie wasting, contribute to the metabolic unbalance occurring in cachexia. It is indeed well known that cachectic patients are characterized by an increase in resting energy expenditure^105^, which means that, even at rest, their metabolism is accelerated^106^.

Since the first attempt to understand cancer cachexia, tumor growth has been pinpointed as the culprit for nutrient subtraction and energy consumption^107^. Accordingly, it has been shown that, during tumor growth, nitrogen balance is managed by the tumor and not by the muscle^108^. However, besides sequestration of nutrients from the tumor, a systemic rewiring of the metabolism takes place during cancer cachexia, indicating that other organs are involved in the metabolic alterations occurring in cancer patients. For instance, the liver has been proposed to contribute to energy wasting in cancer patients^109^, at least in part by the generation of phase 2 proteins linked to the systemic inflammatory state. Not only the liver, but also fat tissue is involved in systemic metabolic wasting. Indeed, systemic inflammation also drives tissue browning, which results in systemic lipolysis and thermogenesis^110^.

The tumor is a main producer of factors triggering metabolic reprograming and wasting, including miRNAs, PTHrP (parathyroid hormone-related protein)^106,111,112^, known to cause hypercalcemia in cancer patients^113^, and D-2-hydroxyglutarate (D2-HG), an oncometabolite that is secreted by leukemia cells as a consequence of mutations of the TCA (tricarboxylic acid/Krebs) cycle enzymes isocitrate dehydrogenase 1 and 2. These mutations occur in some myeloid leukemia patients and result in cardiac contractile dysfunction linked to mitochondrial dysfunction, caused by the increased secretion of D2-HG^114^. Interestingly, comparing acute leukemia (AL) patients with other cancer patients, it has been reported that AL is linked to myocardial dysfunction^115^. Moreover, the rate of AL patients who develop heart failure significantly increases upon chemotherapy^116,117^. Consequently, although leukemia patients do not commonly develop cachexia^118^, the associated cardiac dysfunction may result in an increased susceptibility of AL patients to the development of the wasting syndrome.

Furthermore, tumor growth can directly affect systemic circadian rhythms^119^, an alteration that has been functionally linked to the onset of insulin resistance. Accordingly, cancer cell-induced alterations can eventually affect insulin and glucose metabolism, which per se impact on both cardiac^120^ and skeletal muscle function^121^. Thus, insulin resistance and glucose insensitivity were associated with increased weight loss in cancer patients^122^. For instance, tumor growth negatively affects plasma insulin and glucose levels in cancer-bearing mice^123,124^. As an example, leukemia cells, of mouse models and patients, actively induce insulin resistance by prompting the production of insulin-like growth factor (IGF)-binding protein 1, in order to exclusively exploit glucose availability^123^. Furthermore, cancer cells induce changes of the metabolic profile of other tissues and of the gut microbiome, ultimately conveying insulin resistance and reduction of the anabolic factor IGF-1^3,125^. Moreover, decreased insulin levels have been functionally linked to cardiac wasting, as administration of insulin is able to attenuate cardiac atrophy, while reducing glucose uptake in the tumor^126^, a strategy that might also be important for skeletal muscle. Overall, these findings provide new opportunities for therapeutic interventions aimed at restoring glucose supply in the muscles. Whether this approach may enable to delay or recover cancer cachexia remains to be demonstrated.

Several pieces of evidence recently pinpointed to an altered role of lipid homeostasis in driving skeletal muscle wasting. For instance, it has been shown that wasting skeletal muscles switch to fatty acid oxidation (FAO) as the prominent source of energy production^127,128^. Moreover, FAO has been functionally linked to the wasting phenotype in cancer, and limiting FAO prevents skeletal muscle wasting, either through pharmacological inhibitors^129^ or by genetic inhibition of lipolysis^130^. Whether FAO may be impaired during cardiac wasting has yet to be clarified.

In turn, excessive mitochondrial activity and intermediate overload, caused by increased fatty acid metabolism, may cause an increase in oxidative stress and mitochondrial ROS, eventually leading to dysfunctions^131,132^. Coherently, dysfunctions in mitochondrial metabolism are common alterations occurring in wasting skeletal muscles^133^. Similarly, mitochondrial DNA (mtDNA) released upon stress in cardiac cells can act as a DAMP, and hence as a ligand for TLR9, an endosomal TLR isoform. Activation of TLR9 by mtDNA is responsible for impaired induction of autophagy and the ensuing accumulation of dysfunctional mitochondria and oxidative stress after doxorubicin-induced cardiac injury^87^. Moreover, in skeletal muscle, TLR9 has a key role in coordinating with Beclin1 to activate AMPK under energetic stress^134^. Nevertheless, the role of such an mtDNA-TLR9 axis in cancer-induced cardiac and skeletal muscle atrophy has yet to be evaluated.

Finally, the inflammatory state per se promotes several metabolic alterations, eventually triggering wasting. For instance, inflammatory states, like those occurring during chronic cardiomyopathy or cancer, are known to halt iron uptake by the gut and promote iron retention by macrophages^135^. The resulting iron deficiency triggers anemia, which might further impact on cardiac function and skeletal muscle oxygenation^136^. Interestingly, it has been demonstrated that, at least in the heart, iron-deficient anemia might directly affect the functionality of cardiac cells^137^. In line with this view, several clinical trials in cardiopathic patients have shown that iron supplementation restores cardiac function and muscle strength^138^. Nevertheless, this approach cannot be directly applied to cancer patients as cancer growth itself directly depends on iron supply^139^. Moreover, it has been shown that chemotherapy-induced cardiotoxicity partly depends on excessive accumulation and altered compartmentalization of iron in the heart^140,141^ leading to mitochondrial iron overload and dysfunction.

### The gut microbiota-muscle axis

The gut-associated lymphoid tissue is considered as the largest immune organ of the body. Therefore, it is not surprising that an association between systemic inflammation and gut dysbiosis has been demonstrated in several chronic diseases associated with cachexia, including heart failure^142^. Consistently, a number of studies demonstrated a link between dysbiosis and cardiovascular diseases^143–145^ as well as cancer^146,147^. Accordingly, an increase in intestinal permeability is frequently recognized in cachexia-associated diseases and could facilitate the diffusion of pro-inflammatory molecules across the gut barrier, thus contributing to the systemic inflammatory state^148^.

Mechanistically, besides stimulating the systemic increase of pro-inflammatory cytokines, gut microbiota could lead to muscle wasting by decreasing amino acid bioavailability, by stimulating the TLR/NF-kB pathway through the release of pathogen-associated molecular patterns (PAMPs)^149^, and via the production of cachectic metabolites^104^.

The hypothesis of a systemic signalosome, originating from gut microbiota and targeting distant organs like muscles, was supported by trials showing that modulation of gut microbiota can change immune/inflammatory parameters in cancer patients undergoing esophageal surgery^150^. In line with these findings, interventions on gut microbiota can prolong survival by reducing cancer proliferation, muscle wasting^103^, and fat loss^151^ in mouse models of cancer cachexia. Similar interventions have been proven effective in preventing cardiac atrophy and dysfunction in preclinical models of anthracycline-induced cardiomyopathy^152^. However, experimental proofs of the involvement of gut microbiota in cardiac muscle wasting in cachectic cancer patients are still lacking.

### The impact of chemotherapy on skeletal and heart muscle wasting

Besides chronic tumor-host interactions, acute drug toxicity and long-term side effects of anti-cancer treatments can significantly contribute to chronic muscle wasting in cachexia^153^. Despite a rapid evolution of anti-cancer treatment options, cytotoxic chemotherapy remains the first line and preferred treatment for most cancers. Unfortunately, the presence of cachexia reduces tolerance and response to treatment, activating a futile cycle that eventually reduces the quality of life and survival. In cancer patients, tumor growth might, on the one side, impair the ability of the host to adapt to stress imposed by chemotherapy and, on the other side, directly affect muscle and systemic metabolism^154^. Moreover, most of anti-cancer drugs are severely cardiotoxic^155^, making patient management during cancer treatment and follow-up even more difficult, while increasing the risk of an exacerbation of cachexia.

Chemotherapy itself can contribute to the alteration of the circulating milieu. On the one hand, chemotherapy potentially limits the release of tumor-derived cytokines, therefore relieving cachexia. On the other hand, host tissues may be directly affected by drug toxicity which frequently activates an inflammatory response, thus exacerbating cachexia. For instance, chemotherapy treatment has been shown to trigger GDF15 following endothelial damage^156^. On the same line, the promotion of systemic inflammation might indirectly exacerbate the muscle catabolic action and the systemic dysmetabolism induced by inflammatory molecules, such as TNFα, that is both released by the tumor^157^ and by the host^158^ upon chemotherapy administration.

Moreover, protein hypercatabolism and impaired anabolism are directly affected by both cytotoxic and targeted chemotherapy, further contributing to muscle wasting^159^. In particular, the direct effect of anti-neoplastic drugs on myofibrillar protein degradation and myofiber atrophy has been demonstrated^160,161^. The mechanisms underlying skeletal muscle atrophy in response to chemotherapy are the same as the ones involved in cancer-mediated wasting. In detail, proteasome- and autophagy-mediated protein degradation are induced by cisplatin^162^, cyclophosphamide, doxorubicin, and fluorouracil mixture^163^, or anthracyclines (e.g. doxorubicin) alone^164^.

In the cardiac muscle, the impact of chemotherapy on the main catabolic and anabolic pathways appears even more complex. A comprehensive study, comparing skeletal and cardiac muscle response to doxorubicin, has been performed by two independent research teams. In the first study^64^, albeit similar mass loss was observed in skeletal and cardiac muscles upon doxorubicin exposure, protein synthesis, content in ubiquitinated proteins, and expression of atrogenes were less affected in the heart than in the skeletal muscle. Similar results were shown by the second study^164^, although reporting a controversial activation of autophagy in the skeletal muscle. Albeit, a consensus on the role of autophagy in the cardiac response to anthracyclines has not been reached yet^165^, major studies point to an impairment of the ALP as a major determinant of chemotherapy-induced cardiac atrophy and dysfunction^87,166,167^. Accordingly, anthracycline-induced damage has been associated with failing autophagic clearance of damaged organelles, resulting from the stimulation of TLR9 via mtDNA release by injured cardiomyocytes^87^.

In addition, anthracyclines may directly impact the myofibrillar content in both skeletal and cardiac muscles, further contributing to muscle loss (reviewed in Hiensch et al.^168^). In addition to the previously mentioned role of anthracyclines in regulating metabolism and TLR9 in the skeletal muscle, doxorubicin-induced oxidative stress leads to mitochondrial dysfunction^169–171^, and oxidative modification of myofibrillar proteins, which increases their susceptibility to degradation via calpain‐1 and caspase‐3^172,173^. Moreover, doxorubicin activates all major proteolytic systems, including calpains^173,174^, the UPP^63^, and autophagy^63,175^ in skeletal muscles. Likewise, doxorubicin leads to atrophy also in cardiomyocytes, via activation of MuRF-1^176^ by CDK2-dependent phosphorylation of FoxO1 at Ser-249^177^. Of note, FoxO1 and FoxO3 are potent regulators of muscle atrophy (detailed in Box 4).

Among the proteolytic processes that are induced by doxorubicin in cardiac muscle cells is intracellular activation of matrix metalloproteinase 2 (MMP2), which, in turn, can result in the degradation of both sarcomeric proteins and myofilaments, including titin^178^. Intriguingly, MMP2 is also expressed by skeletal muscle cells, even if at low levels compared to calpain-1^179^, and release of titin from skeletal muscles has been associated with muscle atrophy^180^. Nevertheless, the contribution of MMP2 to proteolysis induced by doxorubicin in tumor-bearing animals has yet to be evaluated.

Both loss and truncation of titin result in skeletal muscle atrophy with reduced strength, severe sarcomere disassembly, and lethality^181,182^. In contrast, impaired titin integrity results in considerably different phenotypes in the heart. Loss of titin leads to dilated cardiomyopathy with systolic and diastolic dysfunction, while titin truncation or deletion of the N2B segment, that impair sarcomeric array, lead to cardiac atrophy with preserved function^182,183^.

Another layer of regulation of titin is provided by the RNA-binding protein known as Quaking, which is downregulated in response to doxorubicin^184^. Quaking inhibits doxorubicin-mediated cardiotoxicity via regulating cardiac circular RNAs, including titin-derived circular RNA in cardiomyocytes. Mechanistically, Quaking deletion in cardiomyocytes increases sensitivity to doxorubicin, whereas its overexpression attenuates doxorubicin-induced cardiac atrophy^184^. Nevertheless, the role of titin degradation in the context of cancer cachexia has yet to be elucidated.

Concomitantly with increased protein degradation, doxorubicin is also responsible for impaired muscle protein synthesis^185^, resulting from the inhibition of the mTOR pathway^185^. Of note, mTORC1 is a major regulator of insulin signaling, however, the disruption of the insulin pathway by doxorubicin has only been detected in skeletal muscles^186^, but not in the heart^187^.

As previously reported, the alteration of energy metabolism, and in particular the occurrence of a systemic energetic failure, is obtaining an increasing consensus as a major cause of cachexia. Whether the energy crisis induced by tumor growth arises from inflammation and mitochondrial dysfunction or from excessive oxidative stress is still debated. Most of anti-cancer drugs enhance oxidative damage in both the skeletal and the cardiac muscle. In the former, oxidative stress can be directly linked to protein hypercatabolism and wasting^160,161^, while in the latter its role has been downscaled, also considering the limited success of anti-oxidants against the cardiotoxicity of drugs like doxorubicin^188^.

Considering metabolic alterations in the skeletal and cardiac muscles, chemotherapy has been shown to partly recapitulate and/or exacerbate cancer-induced muscle alterations^154^, while the cardiac metabolome has been mainly studied with the aim of identifying biomarkers of cardiotoxicity^189^. Instead, only few studies have analyzed tissue-specific alterations of the metabolome during cancer and chemotherapy-associated cachexia. Nevertheless, some common metabolic alterations featured by skeletal and cardiac muscles upon chemotherapy have been identified, which include the increase of free amino acids, likely indicating increased proteolysis, and the reduction in β-oxidation^154,189^. On the contrary, the flux through the TCA cycle is diminished in the skeletal while augmented in the cardiac muscle, potentially as the only mean to sustain the vital function of heart contraction.

BOX 4 FoxO. The forkhead box transcription factors (FoxO) are important for muscle differentiation, metabolism, and atrophy^210^. FoxO1 is key for myoblast differentiation and is, like FoxO3, central for the regulation of muscular atrophy^210^. In addition, FoxO transcription factors act as sensors of metabolic changes. For example, FoxO1 interacts with the promoter of pyruvate dehydrogenase kinase 4 and induces its expression in skeletal muscles after energy deprivation^211^. As a consequence, FoxO1 enables the maintenance of blood glucose levels by inhibiting the pyruvate dehydrogenase complex and the glycolytic flux^211,212^. In a different metabolic context, insulin (as well as IGF1) suppression blunts the activity of PI3K and Akt, which results in the activation of FoxO and the subsequent induction of atrogenes expression, e.g. Atrogin-1 and MuRF-1, in skeletal muscle^213,214^. In the heart, FoxO3 and FoxO1 KO result in myocardial hypertrophy due to reduced atrogenes expression and aberrant activation of Calcineurin phosphatase^215^. In detail, Calcineurin dephosphorylates the transcription factor NFAT (nuclear factor of active T cells), allowing its nuclear translocation and induction of pro-trophic target genes (e.g. α-skeletal actin and β-myosin heavy chain). Conversely, the FoxO target gene Atrogin-1 ubiquitinates and degrades Calcineurin, which further attenuates hypertrophy^216^. Interestingly, in cardiomyocytes, Atrogin-1 acts as a positive feedback regulator of FoxO activity^217^. On the contrary, FoxO-induced inhibition of Calcineurin also blunts its inhibitory function on Akt, leading to an accumulation of phosphorylated (active) Akt, which further can induce hypertrophy^216^. Albeit, FoxO members (in particular FoxO1 and 3) are primarily regarded as inducers of atrophy, their role in cancer progression as well as cancer cachexia still remains to be elucidated.

## Conclusion

Cancer cachexia represents an urgent medical need, due to the great impact on patients’ quality of life and the high penetrance of this condition. Patients with cancer cachexia are often too weak to tolerate standard doses of chemo- and radiotherapy, that may be eventually interrupted, resulting in poor prognosis and increased mortality^190^. Moreover, patients suffering from wasting of diaphragm and/or cardiac muscles often die prematurely because of respiratory and/or cardiac failure^191^. Finally, the cancer itself as well as major anti-cancer treatments have a long-lasting, detrimental effect on myocardial function^192^. It has been shown that cancer survivors have an increased risk of developing cardiac complications, which may manifest even years after cancer clearance and/or completion of oncological treatments^192^, emphasizing the importance to increase our understanding of the link between cancer and cardiac myopathies. The research for molecular drivers of this tremendous and mostly untreatable complication of cancer has been neglected for a long time, as cachexia has been originally linked to reduced food intake. More recently, research on cachexia sparked a novel interest as it is emerging as specifically driven by defined molecular alterations, hence it can be modeled and targeted independently from tumor growth.

While the field of cachexia mainly developed as intertwined with the modeling of skeletal muscle atrophy, cardiac wasting is gaining interest as a major cause of death^191^. Hence, the definition of the mechanism of cardiac wasting holds great potential for the management of cachexia.

The list of inter- and intracellular signaling pathways and molecules presented here is far from being exhaustive, which reflects the rapid development of the field and the complexity of the molecular regulation of cachexia but provides a framework to address the potential analogies between cardiac and muscular wasting. Taken together, inter- and intracellular signaling pathways stand as a central mechanism controlling the autophagy-lysosomal pathway, the ubiquitin-proteasome pathway as well as immunological and metabolic changes during cancer, and integrating the complex phenomenon of cancer cachexia^193^. Further investigations are needed to identify details and differences of cancer-induced cachexia in the skeletal and heart muscle. Consequently, striving for further investigation of the molecular background and the interplay between cancer, metabolism, and cardiac cachexia is essential to improve treatment of cancer patients.
